# Identification of Pathway Deregulation – Gene Expression Based Analysis of Consistent Signal Transduction

**DOI:** 10.1371/journal.pone.0041541

**Published:** 2012-07-25

**Authors:** Jakub Mieczkowski, Karolina Swiatek-Machado, Bozena Kaminska

**Affiliations:** Laboratory of Transcription Regulation, Department of Cell Biology, The Nencki Institute of Experimental Biology, Pasteur 3, Warsaw, Poland; Hemocentro de Ribeirão Preto, HC-FMRP-USP., Brazil

## Abstract

Signaling pathways belong to a complex system of communication that governs cellular processes. They represent signal transduction from an extracellular stimulus via a receptor to intracellular mediators, as well as intracellular interactions. Perturbations in signaling cascade often lead to detrimental changes in cell function and cause many diseases, including cancer. Identification of deregulated pathways may advance the understanding of complex diseases and lead to improvement of therapeutic strategies. We propose Analysis of Consistent Signal Transduction (ACST), a novel method for analysis of signaling pathways. Our method incorporates information regarding pathway topology, as well as data on the position of every gene in each pathway. To preserve gene-gene interactions we use a subject-sampling permutation model to assess the significance of pathway perturbations. We applied our approach to nine independent datasets of global gene expression profiling. The results of ACST, as well as three other methods used to analyze signaling pathways, are presented in the context of biological significance and repeatability among similar, yet independent, datasets. We demonstrate the usefulness of using information of pathway structure as well as genes’ functions in the analysis of signaling pathways. We also show that ACST leads to biologically meaningful results and high repeatability.

## Introduction

Though gene expression microarray-based experiments are popular in life science research, microarray data analysis and interpretation of its results are still challenging. Typically, these analyses lead to lists of genes with the most differential expression between the compared groups. An important limitation of such approach is that genes with moderate but meaningful expression changes may not meet the strict cutoff and alternation of molecular processes may be missed [1], [2]. These are particularly important when studying a complex disease, for instance cancer, that is associated with changes of expression of multiple genes. Moreover, frequently there is little overlap between lists of genes obtained by different groups exploring the same biological conditions [3].

These limitations could be overcome by signaling pathway analysis. Signaling pathways are maps of processes occurring in cells and may represent signal transduction from an extracellular stimulus via a receptor to intracellular mediators, as well as intracellular interactions. Various studies have demonstrated the potential of using gene expression profiles for the analysis of oncogenic pathways [4]. Furthermore, better understanding of pathways deregulations may lead to improvement of cancer therapeutic strategies [5].

Signaling pathways contain information not only about the presence of a given element (gene or protein), but also about interactions between their elements e.g. co-regulation. All elements in a particular pathway have their specific functions and positions in a given pathway, and both of them depend on the pathway. Furthermore, a given interaction may represent protein-protein relationship (e.g. phosphorylation) or gene-gene relationship (e.g. indicating relation of transcription factor and target gene product) [6].

Basically, there are two different types of methods for signaling pathway analysis. One of them [7]–[10] attempts to detect co-expressed genes and reconstruct a pathway structure. The second group of methods uses externally defined pathway definitions to detect altered pathways in underlying constituents either in pathways of a given length [11] or in all of the pathways 6,12. In this study we focused on the latter approach. An interesting review of evolution and limitations of such methods was recently published by Khatri et al [13].

One of the methods for analysis of signaling pathways is Signaling Pathway Impact Analysis (SPIA) proposed by Tarca et al. [6]. This method considers overrepresentation of differentially expressed genes, as well as the function of every gene in a given pathway and the magnitude of gene expression changes. It is important to notice that SPIA uses an assumption that the closer a gene is situated to the beginning of the pathway, the greater impact on the signal transduction it has. To score each signaling pathway SPIA uses two independent factors: a test for overrepresentation of differentially expressed genes in a given pathway and perturbation of a given pathway measured by propagating expression changes across the pathway. The latter is computed with the bootstrap procedure with a gene sampling model.

Another method, Bayesian Pathway Analysis (BPA) presented by Isci et al. [12], employs Bayesian network models. Using Bayesian network models allows us to capture linear as well as non-linear interactions between genes and emphasize only strong relations in the observed data. BPA models each pathway as a Bayesian network and quantifies the degree to which observed experimental data fit to a modeled network. The significance of each pathway is estimated by applying randomization via bootstrapping.

In this paper we propose Analysis of Consistent Signal Transduction (ACST), a novel method for the analysis of signaling pathways. ACST incorporates three types of features: pathway topology information, the position of every gene within a given pathway and the magnitude of changes of all analyzed genes. ACST requires neither arbitrary cut-offs nor arbitrary parameters. In contrast to previously published methods, we propose a method without an oversimplifying assumption that gene expression changes unambiguously reflect protein action (e.g. phosphorylation). In our method each pathway is examined for a consistent relationship between transcription factor and its target genes-genes that are directly regulated by this transcription factor. Expression changes of the latter components of the examined pathway reflect changes in physiological events. Our results are evaluated in forms of nominal p-values and false discovery rate correcting for multiple hypothesis.

While testing ACST not only did we use a number of independent experimental datasets, but we also tested repeatability of results examining biologically similar, yet independent, datasets. We compared ACST with results achieved with the methods mentioned above (SPIA and BPA) and with a popular method designed to gene set analysis: Gene Set Enrichment Analysis [1]. Comparison with the last method allowed us to test the utility of incorporating information of pathways structure.

## Materials and Methods

In order to apply the Analysis of Consistent Signal Transduction we need (i) gene expression measurements and (ii) definitions of signaling pathways. (i) can be obtained with gene expression microarrays. (ii) may be defined using KEGG [14], Biocarta [15] or any other pathway database which contains information about relation of transcription factor and target gene product (for details see subsection Pathways structure and its implications). In our study we used definitions from KEGG.

### Definitions and Notations

In this study a particular signaling pathway is represented as a directed graph 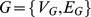, where the nodes 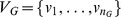 represent genes while directed edges 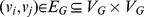 represent relations between respective genes. 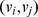 is a direct edge which starts in 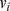 and ends in. 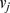 A path 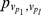 in a directed graph 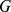 is a sequence 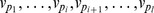, such that for each 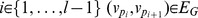 and for every 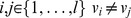, if 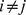. The length of a path 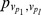 is identified with the number of elements in 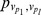 and is denoted as 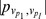. The node 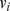 is a leaf in graph 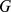 if and only if 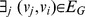 but . 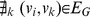 The 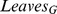 stands for a set of leaves of graph 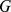. By a subgraph of graph 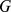 we understand a graph 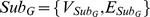 such that 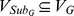 and 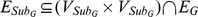. Let 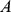 and 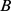 be subgraphs of graph G. A directed distance in graph 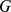 from 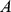 to 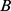 is given by 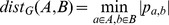 if any 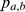 exists, and 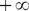 otherwise. In other words, a directed distance from 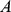 to 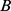 is equal to the number of nodes in the shortest path which starts in node from 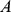 and ends in node from 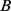. If 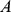 and 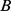 overlap, 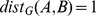.

**Figure 1:**
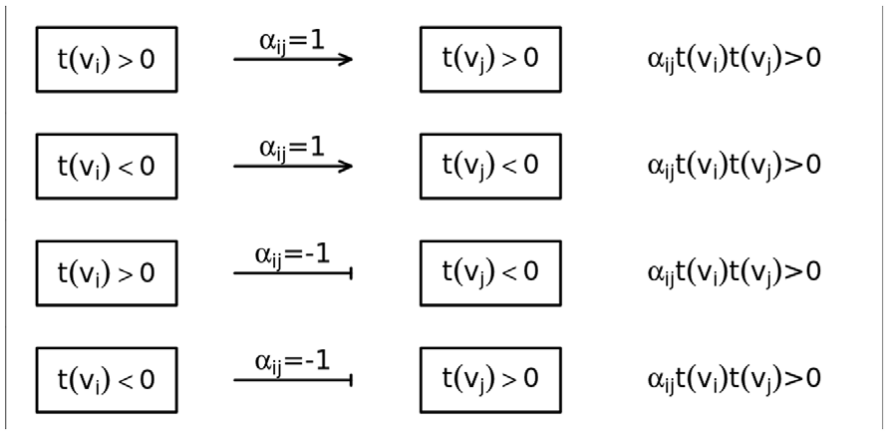
The plot presents all consistent relations and stand for any sign statistics which reflect the direction of expression changes between the analyzed conditions of genes and expresses the type of interaction.

For the purposes of this study we need to define a consistent relation and a consistent graph. Assuming that two experimental conditions (e.g. disease vs. control) are compared, 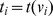 stands for sign statistic (e.g. t-test, logFoldChange). The sign of this statistic must determine the direction of expression changes between the analyzed conditions of gene 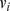. We say that 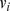 is in consistent relations with 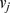 if and only if 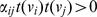, where 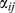 reflects the type of interaction 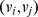. 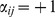 if 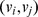 stands for activation, 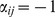 if 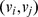 stands for repression and 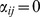 otherwise. Note that 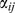 reflects the real biological interactions between genes 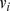 and 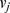 and is given by the chosen signaling pathway database. Figure 1 presents four possible consistent relations.

A consistent graph is a graph in which all edges represent consistent relations. Moreover, a consistent subgraph 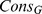 of graph 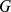 is maximal if and only if

1. 
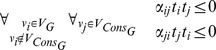



2. 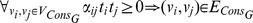
where 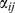 reflects the real biological interactions and is given by a particular pathway database.

### Description of ACST

In general, ACST shares the scheme presented by [16], in which two statistics are computed. The first, a local statistic, is computed for each gene. The second statistic, a global statistic, is computed for each analyzed pathway and is based on local statistics of genes which belong to a particular pathway. The score of global statistics is assessed with a permutation model (subject sampling model) followed by an estimator of false discovery rate (FDR) studied in [17] and indicated in [16]. Although class label permutations allow us to maintain gene-gene relations in each sample, Efron and Tibschirani [18] showed that the permutation model leads to smaller scores than scores computed with original labels. In order to enable comparison of pathway scores from original data and permutation model we used standardization.

#### Local statistic

To compute local statistic we use two-sided t statistic, 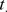, with the Welch modification. Let 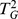 stand for a set of squared t statistics computed for all genes from the chosen pathway database. A local statistic for a given gene 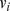 is
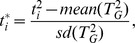
where 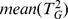 and 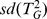 stand for mean and standard deviation of values in 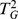. Similarly to [18], we noticed that the permutation model leads to smaller t-statistics than t-statistics computed with original labels. In order to enable comparison between original data and permutation model we used standardization (for details see [18]).

#### Global statistic

**Figure 2:**
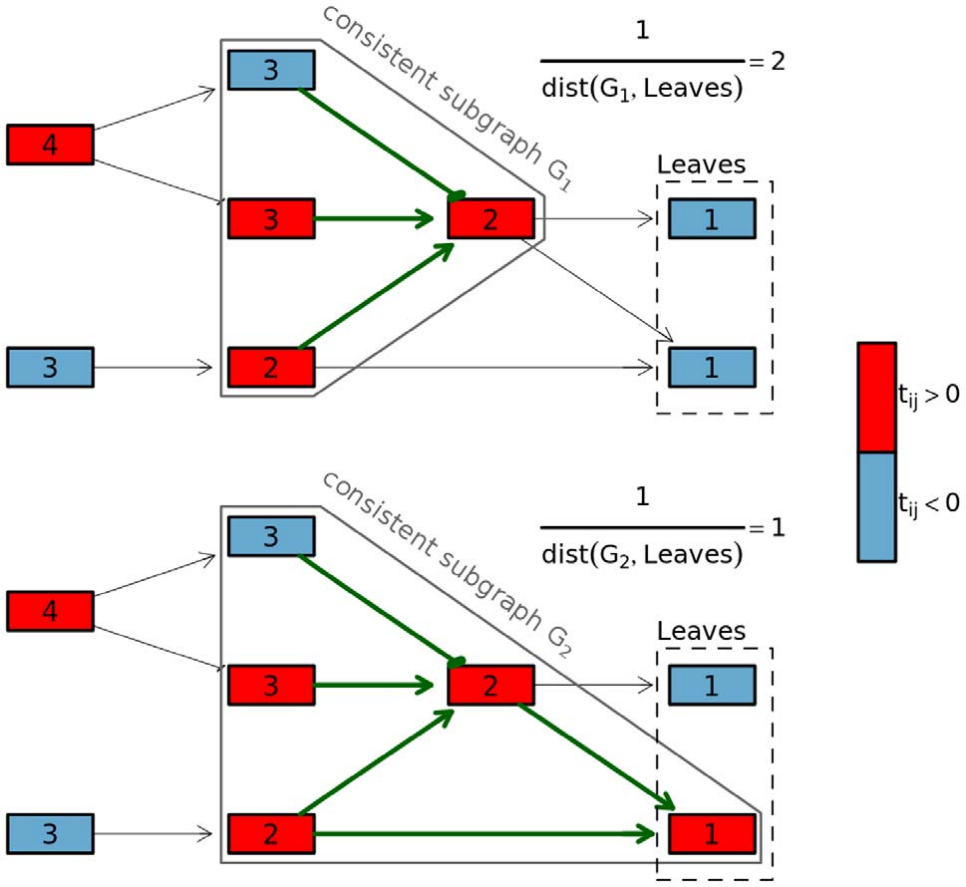
The plot presents scores of positions of found consistent subgraphs. Both figures present the same artificial graph but with marked different expression changes. The expression changes were marked with colors. The red color marks overexpression in the tested group (with regard to control), while the blue color represents underexpression. The nodes are marked with their distance (see the definitions and notations subsection) to leaves of the graph. The green arrows represent consistent relations.

To compute global statistic each graph 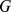 is searched for maximal consistent subgraphs. Please note that in this step we use non-standardized t statistics, i.e. 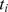, because their signs reflect the real direction of changes. If any maximal consistent subgraph 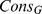 is found, its score 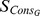 is calculated as follows:
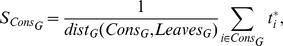
where 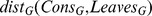 is a distance from 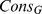 to 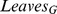. Using 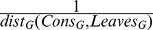 we weigh the sums of standardized t statistics with respect to the distance between the found subgraphs and leaves of the searched graph. Figure 2 presents this dependency. The global statistic for graph 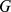, 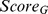, is a sum of scores of all maximal consistent subgraphs found in 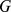. If no consistent subgraph is found in 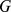 the 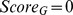.

The occurrence of a consistent subgraph stands for relative (with reference to control) change of gene-gene co-regulations in tested biological conditions. With regard to biological knowledge we scored only maximal consistent subgraphs. Using only maximal consistent subgraph does not necessarily lead to the highest score, but the greater range of transduction is found, the more likely it is that a functional change is observed. Additionally, since we do not expect that the majority of all analyzed genes are differentially expressed, negative 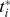 means that the direction of expression change of gene 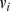 might be random as well as its presence in a given consistent subgraph.

The 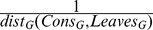 scores for the place where given co-regulations are found in the considered pathway. Using such factor we give a higher score to the co-regulations found at the end of the pathways. We do so because co-regulations at the beginning of a pathway not necessarily induce a specific cellular response, because perturbation of downstream genes may impact the process of signal conversion. Since the response (cellular processes such as proliferation or migration) is accompanied with downstream activation we put more emphasis on the genes that are at the end of the pathway.

To assess the global statistic we used class label permutations. Class label permutations allowed us to maintain gene-gene relations in each sample and draw conclusions about biological replications. Let 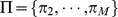 be a random sample of permissible permutations of class labels and 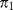 be the permutation corresponding to the observed class labels. Then, the empirical p-value for the graph 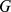 is computed as:
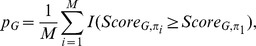
where 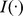 is an indicator function. For the purposes of this study we used 1000 permutations for each dataset. To estimate false discovery rate associated with the selected threshold for significance we used a ‘resampling-based point estimator’ of FDR [17]. This estimate of FDR was developed for controlling FDR when conducting multiple dependent tests. An R code of ACST is available at http://jmieczkowski.nencki.gov.pljmieczkowski.nencki.gov.pl and in Additional file S1.

Considering the methodological challenges raised in [13] we believe that ACST can be easily transformed to analyze dynamic response in tested conditions. The main goal of our method is to identify subgraphs in which genes expression changes are consistent with a particular pathway definition. Thus, if proper data are obtained, it would be easy to look at the dynamic changes in the found subgraphs (i.e. number of nodes, score of consistency etc.).

### Pathways Structure and its Implications

Pathway definitions contain information not only about gene expression interactions, but also on protein-protein interactions (e.g. phosphorylation). However, gene expression data only provide information regarding mRNA levels in analyzed samples. Therefore, in our work we used only information regarding gene expression interactions (marked GErel in KEGG). This type of interaction describes the relationship between particular transcription factor and the protein whose expression is directly regulated by this transcription factor. The proposed algorithm does not include other type of protein action, especially enzymatic reaction like phosphorylation as they are not reflected by microarray data. Due to limitation to genegene relations in this study we analyzed only 47 signaling pathways out of 209 from KEGG database (others do not contain gene-gene relations, for a detailed list of analyzed pathways see Additional File S2). We realize that such limitation of applicability might be seen as a drawback, but we believe that it is a drawback due to type of data, not of the method itself. Thus, in contrast to other signaling pathway methods we deliberately do not make assumptions about protein-protein interactions based on transcriptional expression data. For instance, we believe that it may be misleading to conclude about activation by phosphorylation based solely on transcriptional expression profiles.

Another innovative aspect of our method is looking for consistent relations. We agree that it is rather restrictive and on some occasions our method may miss some pathways. However, occurrence of consistently related genes is of great importance. Indicating signaling pathway in which such consistent relation occurs might be very useful for understanding the biological process as well as for identifying targets for molecular therapy. We think that the methods which lead to a lower number of false positives are desired, even if some true positives might be missed. Nevertheless, it is important to note that ACST may simply be used also with protein expression data (e.g. data from protein expression arrays). Since the t-test does not depend on scale, some gene expression data may simply be merged or replaced with proteomic expression data and more types of interactions may be used.

### Selection of Datasets

Assessing performance of any new method for pathway analysis is difficult because of the absence of gold standard [1], [6]. The best way to evaluate a new method is to reanalyze biologically well-known conditions and to compare obtained results with the results of already existing methods. Testing our method we decided to use one more criterion-repeatability of results. As pointed in [13] biological data may be affected by several confounding factors, so we believe that obtaining repeatable results on independent data sets is strongly desirable. Considering our goal to obtain correct and biologically meaningful results we chose only datasets for which results in original papers were confirmed using biochemical methods or/and from well-studied biological cases. To evaluate the repeatability we used Spearman’s rank correlation. To assess significance of computed correlations we used F-test computed as follows 
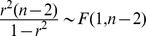
, where 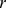 is a correlation coefficient, 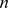 is a number of correlated values and 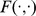 stands for F distribution. The Bonferroni correction was used to determine the significance of the correlations.

In this study we used nine publicly available datasets. All the raw data were downloaded from GEO database [19] and the used datasets are marked by GSE4107 [20], GSE9348 [21], GSE24514 [22], GSE8671 [23], GSE15641 [24], GSE14762 [25], GSE9574 [26], GSE10797 [27] and GSE5563 [28]. The GSE4107 dataset had already been used by [6]. Four of collected datasets (GSE4107, GSE9348, GSE24514 and GSE8671) were collected to compare gene expression in colorectal cancer and control samples. In our analyses these sets are marked CC1, CC2, CCms and CCade respectively. The next two datasets (GSE15641 and GSE14762) were collected to compare gene expression in renal cell cancer and control samples. Originally the GSE15641 dataset was employed to compare different subtypes of renal cell cancer and normal samples, but in our study we just used cRCC (clear cell RCC also used in GSE14762) and normal samples. We marked this set with RCC1, and the GSE14762 dataset with RCC2. Other two datasets (GSE9574 and GSE10797) were collected to compare gene expression in epithelial breast cells from breast cancer patients and control group. These sets were marked BC1 and BC2 respectively. Finally, the GSE5563 dataset was collected to compare gene expression in Vulvar Intraepithelial Neoplasia and control samples. We marked this set VIN. Referring to methodological challenges presented by Khatri et al. [13] we believe that the presented criteria for selection of groups of datasets may be used as a benchmark for other methods of signaling pathway analysis.

### Preprocessing and Analysis of Microarray Data

All data manipulations were performed using R statistical environment [29] and relevant Bioconductor software [30]. According to suggestions in [31] we applied quality analyses using *affyQCReport* Bioconductor package [32]. The quality of the majority of the downloaded CEL files was acceptable, but some of them did not meet the basic quality criteria (GAPDH 3′:5′ ratio 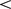
1.25, 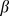-actin 3′:5′ ratio 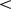
3 and scale factor within 3-fold of the mean of all chips) and these microarray were eliminated from further analyses (see Additional File S2). All the microarray datasets were preprocessed with GC-RMA separately. Next, in order to filter out unreliably measured genes and to uniquely map probe sets to genes we applied filtering and mapping procedures described in [33]. Briefly, after preprocessing we applied (i) Present/Absent/Marginal filtration to verify specificity of measured signal and (ii) transformation of probe set measurements into gene measurements, excluding probe sets that are annotated to more than one gene (for details see [33]). To do so, we used annotations of probe sets to genes provided in the Ensembl database.

In order to compute Signaling Pathway Impact Analysis we used *SPIA* and *limma* (genes with adj.p-value
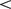
0.05 were considered significantly differentially expressed) Bioconductor packages. Gene Set Enrichment Analysis and Bayesian Pathway Analysis were performed with java codes provided by their authors. Our method, Analysis of Signal Transduction, was implemented under R statistical environment and to import pathways definitions we used *parseKGML2Graph* function from *KEGGgraph* package and we used only nodes that represent genes. SPIA, BPA and GSEA were used with default settings. By default these methods test different number of signaling pathways, i.e. SPIA tested 128, BPA 206 and GSEA 186. To facilitate comparison of repeatability of tested methods for SPIA, BPA and GSEA we reported results obtained for all tested pathways as well as only for pathways used by ACST (with recomputed adjusted p-values). For all presented methods, pathways with adjusted p-value below 0.25 were considered significant.

## Results and Discussion

In the present study we analyzed nine publicly available datasets (described below), for which considerable background information is available. All of them have been used to address biological questions, namely to compare gene expression in disease versus normal tissues. Using real datasets allows us to preserve the gene-gene correlations and dependencies. All the methods have been validated by testing: (i) whether the obtained results confirm biological knowledge concerning gene expression deregulation under studied conditions, and (ii) whether the obtained results are repeatable on similar datasets. In order to verify (i) we used datasets for which the original results of analyzes were further confirmed using biochemical methods. This type of validation is considered the most proper assessment of quality of a novel approach and it was used in previous studies [1], [6]. For verification of (ii) we used three similar, yet independently generated datasets (two of them from the same laboratory).

Additionally, ACTS was compared with several existing methods including the most widely used method for gene sets analysis-GSEA and two methods for pathway analysis-SPIA and BPA. Signaling pathways with adj.p-val
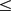
0.25 were considered significant for all tested methods and only these pathways are listed.

### Comparison of Four Colorectal Cancer Datasets

In order to test the repeatability of signaling pathway analyses, we reanalyzed data from three recent studies of colorectal cancers. The first dataset consists of gene expression profiling of samples from young patients (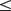
50 years old) with early onset colorectal cancers (n = 12) versus healthy controls (n = 10) [20]. The second dataset consists of gene expression profiling of colorectal cancer samples (n = 24) from older patients (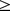
50 years old) and samples from healthy donors (n = 7) [21]. The third dataset consists of gene expression profiling of colorectal cancer sample with microsatellite instability (n = 34) and normal colonic mucosa (n = 15) [22] The fourth data set compared the gene expression profiling of colorectal adenomas (n = 32) with normal mucosa (n = 32) [23]. The results of analysis of colorectal cancer datasets are presented in Table 1.

**Table 1:** ACST results on four colorectal cancer datasets.

CC1			CC2			CCms			CCade		
Name	pv	adj.pv	Name	pv	adj.pv	Name	pv	adj.pv	Name	pv	adj.p
Colorectal canc	0.001	0.013	Colorectalcanc	0.005	0.095	Colorectalcanc	0.014	0.116	Melanoma	0.005	0.093
MAPK sigpath	0.001	0.013	*Cell cycle*	*0.012*	*0.106*	*TLR sig path*	*0.014*	*0.116*	Insulin sig path	0.013	0.131
*TLR sig path*	*0.002*	*0.019*	*Epith cell sig*	*0.013*	*0.106*	*Melanoma*	*0.015*	*0.116*	Mineral abs	0.019	0.131
Prion diseases	0.003	0.023	Shigellosis	0.014	0.106	*Endomet canc*	*0.018*	*0.116*	*Cell cycle*	*0.020*	*0.131*
Bile secretion	0.005	0.033	*Endomet canc*	*0.019*	*0.106*	*Thyroid canc*	*0.018*	*0.116*	Aldoste-reg sod	0.024	0.131
*Epith cell sig*	*0.008*	*0.045*	*Thyroid canc*	*0.019*	*0.106*	African trypanos	0.020	0.116	**Colorectal canc**	**0.030**	**0.131**
Focal adhesion	0.016	0.078	*Wnt sig path*	*0.023*	*0.113*				*Endomet canc*	*0.032*	*0.131*
*Wnt sig path*	*0.023*	*0.101*	*S cell lung canc*	*0.038*	*0.166*				*Thyroid canc*	*0.032*	*0.131*
			Acute myel leuk	0.050	0.199				Basal cell carc	0.032	0.131
									Hedgehog sigpath	0.036	0.134
									*S cell lung cancer*	*0.048*	*0.164*

The table presents results of ACST on four colorectal cancer datasets. Only significantly altered pathways (adj.p-value<0.25) are presented. Signaling pathways indicated for all datasets are written in **bold** while signaling pathway indicated more than once are written in *italic*. Epith cell sig stands for Epithelial cell signaling in Helicobacter pylori infection, S cell lung canc for Small cell lung cancer, African trypanos for African trypanosomiasis, Mineral abs for Mineral absorption and Aldoste-reg sod for Aldosterone-regulated sodium reabsorption.

The utility of the proposed method was tested in terms of its ability to identify the colorectal cancer pathway itself. In all tested datasets ACST identified *Colorectal cancer* pathway. *Wnt signaling pathway*, *Toll-like receptor signaling pathway*, *Epithelial cell signaling in Helicobacter pylori infection*, *Endometrial cancer* and *Thyroid cancer*, *Melanoma* and *Small cell lung carcionoma* were identified for at least two datasets. All of them are fundamental for colorectal carcinogenesis. *Wnt signaling pathway* (identified with adj.p-val
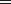
0.009, 0.237 in CC1 and CC2 respectively) is integral to colorectal cancer and more than 90% of patients show alterations that affect it [34]. The importance of *Wnt signaling pathway* in carcinogenesis is well supported by many clinical and experimental studies, and its blockade may lead to new treatment strategies in cancer [35]. In CC1 and CCms ACST also identified *TLR signaling pathway* as significantly perturbed (adj.p-val
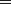
0.009 and adj.p-val
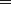
0.15 in CC1 and CCms respectively). TLR signaling plays a crucial role in inflammation, host defense and tissue repair, and a growing body of evidence shows involvement of *TLR signaling pathway* in progression of gastrointestinal tumors, including colorectal cancers [36], [37]. Recent studies have demonstrated that TLR signaling stabilizes the c-Myc oncoprotein through activation of ERK and promotes tumor development [38]. On the basis of these studies, future strategies for treatment of colorectal cancer could involve local intestinal inhibition of TLRs [39].

Four cancer related pathways: *Endometrial cancer*, *Thyroid cancer*, *Melanoma and Small cell lung carcionoma* were highly ranked by ACST in the colorectal cancer tested datasets. The cancers connected with indicated pathways share the same genetic alterations as colorectal cancer e.g. the presence of oncogenic KRAS and/or 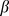-catenin or p53 mutations are common for these tumors [40], [41]. Additionally ACST also indicated a *Epithelial cell signaling in H.* *pylori infection pathway* for CC1 and CC2 (adj.p-val
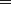
0.128, 0.177 in the first and the second dataset respectively). Although authors of the original work do not provide information about status of Helicobacter pylori infection of analyzed patients it is generally known that in colorectal cancer there is an increased risk of Helicobacter pylori infection [42]. Biological interpretation of significantly changed pathway in the colorectal cancer datasets leads us to the conclusion that ACST is able to detect pathways characteristic for a pathological condition.

**Figure 3:**
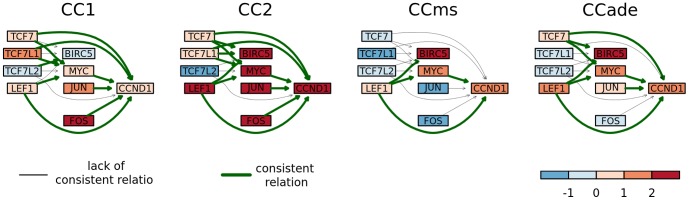
Fragment of *Colorectal cancer* pathway presenting consistent subgraphs of deregulated pathways found for four colorectal cancer datasets. The red color marks a higher expression in cancer samples, while the blue color represents a higher expression in a control group. The color scale represents the magnitude of changes. The green arrows represent consistent relations.

Besides identification of significantly perturbed pathways, ACST allowed us to identify a group of transcription factors and their products that are consistently changed in a particular pathway. The scheme of consistently changed gene expression in identified subgraph in *Colorectal cancer* pathway for all four tested datasets is presented in Figure 3. The common motif for all four colorectal cancer datasets consists of LEF1, Myc and CCnd1 and expression of these genes is higher in cancer patients. Signal transduction by LEF1 and Myc to Ccnd1 may be a driving force of uncontrolled proliferation as Ccnd1 belongs to the Cyclin D family and is required for the cell cycle G1/S transition. We also observed differences among consistent subgraphs identified in the analyzed datasets. Our findings show that in a particular group of clinically related colorectal cancer patients we were able to indicate a group of genes that could be directly responsible for a pathological hallmark. Identification of major factors involved in cancer progression characteristic for a particular group of patients allows us to create a clinically useful hypothesis and indicated potential targets for molecular therapy.

### Application to Other Datasets

In order to further demonstrate the utility of our method, we applied ACST to five additional datasets. The first two datasets originated from the comparison of gene expression changes in renal cell carcinoma with normal kidney tissue samples [24], [25]. Table 2 presents the results obtained with ACST. For these datasets ACST revealed two common significantly perturbed signaling pathways: *Endocrine and other factor regulated calcium reabsorption* and *Vasopressin regulated water reabsorption* (with adj.p-val = 0.048; 0.074 for RCC1 and adj. p-val = 0.148; 0.236 for RCC2 respectively). These two pathways characterize kidney functions and perturbations in those pathways are typical for kidney failure. Vasopressin is a critical regulator of water homeostasis by controlling the water movement from lumen to the interstitium for water reabsorption and adjusting the urinary water excretion. Regulation of calcium homeostasis by reabsorption is one of the main kidney functions and elevated calcium level is one the first symptoms of renal carcinoma. The results are consistent with biological knowledge concerning the pathological state.

**Table 2:** ACST results on Renal Cell Cancer.

RCC1			RCC2		
Name	pv	adj.pv	Name	pv	adj.pv
**Endocrine and other factor-reg calcium**	**0.003**	**0.048**	Aldosterone-reg sodium reab	0.001	0.019
Insulin signaling pathway	0.004	0.048	**Endocrine and other factor-reg calcium**	**0.011**	**0.148**
**Vasopressin-reg water reab**	**0.008**	**0.074**	**Vasopressin-reg water reab**	**0.023**	**0.236**

The table presents results of ACST on RCC datasets. Only significantly altered pathways (adj.p-value<0.25) are presented. Signaling pathways indicated for both datasets are written in **bold**. Endocrine and other factor-reg calcium stands for Endocrine and other factor-regulated calcium reabsorption, Aldosterone-reg sodium reab stands for Aldosterone-regulated sodium reabsorption, and Vasopressin-reg water reab stands for Vasopressin-regulated water reabsorption.

As previously mentioned the main advantage of the proposed method is a possibility to identify the consistent subgraph within the identified pathway. The consistent subgraph is composed of transcription factors as well as proteins that are directly regulated by these transcription factors provided that observed changes have a consistent direction. The subgraph within pathway *Endocrine and other factor regulated calcium reabsorption* is composed of four important protein VDR, ESR1, CALB1 and ATP2B1. VDR and ESR1 as transcription factors regulate the expression of protein which play a critical role in intracellular calcium homeostasis like CALB1 and ATP2B1. An independent study showed that inadequate calcium intake is an important risk factor for various types of cancer. VDR and all vitamin D metabolizing enzymes are expressed in normal kidney but during the malignant transformation, expression of VDR and the metabolizing enzymes are lost, though the implications of this loss are unknown [43]. The identified subgraph within pathway *Vasopressin regulated water reabsorption* is composed of CREB3L2 and AQP2. CREB3L2 is a transcriptional activator that regulates the expression of AQP2-a water channel protein located in the kidney collecting tubule. AQP2 is associated with the pathophysiology of several sodium and water balance disorders [44] (for the consistent subgraphs from pathways called significant in both RCC data see Additional File S3).

Additionally, *Insulin signaling pathway* and *Aldosterone regulated calcium reabsorption* were indicated for RCC1 and RCC2, respectively. Aldosterone regulated calcium reabsorption similarly to the two mentioned pathways describing kidney functions. Multiple lines of evidence implicate the Insulin signaling in the development and progression of cancer. In the context of developing new treatment for renal cell carcinoma, IGF1 signaling has been shown to regulate HIF1-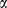, which is a master regulator of hypoxia inducible genes including VEGFR, PDGFR, and TGF-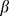 all of which play important roles in the development of RCC [45]. The results obtained on all datasets show that ACST has the power to find a significant pathway.

The next two datasets [26], [27] were collected to compare gene expression in epithelial breast cells from breast cancer patients (14 and 27 in BC1 and BC2 respectively) and control (15 and 5 in BC1 and BC2 respectively) samples. The results obtained with ACST are presented in Table 3. ACST indicated four common significantly perturbed pathways: *TLR signaling pathway* (with adj.p val = 0.009; 0.088 in BC1 and BC2 respectively) *Legionellosis* (with adj.p val = 0.196; 0.015 in BC1 and BC2 respectively) *Wnt signaling pathway* (with adj.p val = 0.022; 0.075 in BC1 and BC2 respectively) *Focal adhesion* (with adj.p val = 0.077; 0.037 in BC1 and BC2 respectively). The indicated pathway reflect the pathological condition. Toll-like receptors (TLR), key receptors in innate immunity, play a role in cancer progression and development, including breast cancer. Signaling pathways like *Wnt signaling pathway* and *Focal adhesion*, are misexpressed in breast cancer and correlate with poor clinical outcomes. It was recently showed that mammary epithelial-specific disruption of focal adhesion kinase retards tumor formation and metastasis in a mouse model of human breast cancer [46]. The presence of *Legionellosis* in this comparison is due to the presence of transcription factor Myd88. Myd88 regulates activation of numerous proinflammatory genes during infection as well as tumor development [47]. As described for previous datasets also within pathways identified for BC datasets ACST indicated substantial subgraphs which in our opinion represent major changes describing pathological condition (for the consistent subgraphs from pathways called significant in both BC data see Additional File S3).

**Table 3:** ACST results on Breast Cancer.

BC1			BC2		
Name	pv	adj.pv	Name	pv	adj.pv
**TLR signaling pathway**	**0.001**	**0.009**	**Legionellosis**	**0.001**	**0.015**
MAPK signaling pathway	0.001	0.009	**Wnt signaling pathway**	**0.002**	**0.022**
Epithelial cell signaling in Helicobacter pylori infection	0.001	0.009	**Focal adhesion**	**0.009**	**0.077**
**Focal adhesion**	**0.005**	**0.037**	**TLR signaling pathway**	**0.014**	**0.088**
**Wnt signaling pathway**	**0.012**	**0.075**	Prion diseases	0.017	0.088
**Legionellosis**	**0.036**	**0.196**	Endometrial cancer	0.020	0.088

The table presents results of ACST on BreastCancer dataset. Only significantly altered pathways (adj.p-value<0.25) are presented. Signaling pathways indicated for both datasets are written in **bold**.

The last dataset, VIN, was generated to compare gene expression profiles between vulvar intraepithelial neoplasia (n = 10) and control samples (n = 9) [28]. Results for that dataset calculated with ACST are shown in Table 4. ACST identified *Cell cycle* and *Melanogenesis* pathways as the most affected pathways (with adj.p val
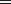
0.017, 0.138 respectively). The importance of *Cell cycle* pathway is well supported by original research [28]. Immunostaining for a proliferation marker Ki67 confirmed differences in *Cell cycle* pathway, revealing considerably higher Ki67 expression in neoplastic tissues as compared to control samples. Perturbation in *Cell cycle* pathway confirms the authors’ hypothesis that vulvar intraepithelial neoplasia, a premalignant disorder, already displays several hallmarks of cancer. The consistent subgraph identified within *Cell cycle* pathway contains many genes (*TP53, CcnE1, CcnE2, CcnA1, Myc*) that are directly responsible for up-regulation of proliferation. The second identified pathway, *Melanogenesis*, has not been associated so far with VIN. However, MITF-one of the genes being part of a consistent subgraph-has been shown recently to act coordinately with transcription factor FoxO to regulate expression of proapoptotic and cell cycle control genes by phosphatidylinositol 3-kinase/Akt/glycogen synthase kinase 3 signaling [48]. This result might indicate a novel player in VIN progression.

**Table 4:** ACST results on VIN.

VIN		
Name	pv	adj.pv
Cell cycle	0.001	0.017
Melanogenesis	0.009	0.116

The table presents results of ACST on VIN dataset. Only significantly altered pathways (adj.p-value<0.25) are presented.

### Repeatability of Tested Methods and Comparison with Other Methods

The five mentioned datasets were also analyzed with SPIA, BPA and GSEA. The results of these analyses are presented in Additional File S2. Although SPIA, BPA and ACST were designed to analyze signaling pathways all those methods are based on different assumptions. SPIA measures overrepresentation of differentially expressed genes and propagation of expression changes, BPA uses Bayesian network models while ACST is designed to capture genes with consistent expression changes. On the other hand, GSEA, which is designed for analysis of gene sets, does not take into account relations among genes and measures enrichment of genes from a tested gene set in the list of differentially expressed genes. Moreover, SPIA and BPA use information not only about gene expression interactions, but also about protein-protein interactions. The different approaches cause that not all KEGG pathways could be analyzed with those methods. BPA may be used to analyze 206 out of 209 pathways included in KEGG database. SPIA analyzes 113 pathways. Since ACST is focused on gene expression interaction it may analyze 47 KEGG pathways.

Importantly, only ACST identified *Colorectal cancer* pathway as significantly perturbed in three colorectal cancer datasets. None of the other methods indicated this pathway as significantly changed, even when computations of adj.p-values were limited to pathways analyzed with ACST. It is worth noticing that regardless of the number of tested pathways, the pathways which seem to be fundamental for colorectal carcinogenesis (in particular *Wnt signaling pathway*, *TLR signaling pathway* or *MAPK signaling pathway*) were not indicated by BPA, SPIA or GSEA. Most of the pathways indicated by GSEA and BPA in colorectal cancer datasets as well as in RCC, BC and VIN characterize cell metabolism (Metabolic pathways are poorly reflected by gene expression interactions and have not been analyzed with ACST). It is generally accepted that cancer cells display dramatically altered metabolic circuitry [49] and it has been shown that reprogramming of a few metabolic pathways indicated by GSEA and BPA is associated with tumor progression. SPIA did not identify any pathway common for all four colorectal cancer datasets but three pathways, *Extracellular matrix*-*receptor interaction*, *Focal adhesion* and *Cell cycle*, indicated for at least two colorectal cancer datasets known to be relevant to colorectal cancer.

To assess repeatability of a given method, we compared an each pair of the results with two factors (i) the number of signaling pathways called significant for both datasets and (ii) Spearman’s rank correlation coefficient between lists of nominal p-values. (i) allowed us to compare agreement of the top of the lists, which is the most important but depends on the cutoff, while with (ii) we measured agreement of the whole rankings. We show values computed for all possible signaling pathways (in brackets) as well as only for the pathways used by ACST (the results are presented in Additional file S3).

For colorectal cancer datasets the highest repeatability was obtained with BPA analyzing all pathways from KEGG. ACST also led to high Spearman’s correlations between colorectal cancer datasets. Although Spearman correlation for CC1 with other datasets is much lower (19 with CCms and 19 with CCade), two pathways significantly perturbed in both sets were indicated. The low correlation could be explained by the fact that in CC1 morphologically normal-appearing mucosa from patients with cancer were compared with that of healthy controls. Our data suggest that the mucosa of patients were actually not normal but were already “primed” for carcinogenesis although it is not cancer tissue. Moreover, only tumors classified as microsatellite stable were included in CC1, while CCms contains only tumors classified as microsatellite unstable. It was shown that patients exhibiting high microsatellite instability have different expression profiles and cancer etiology [50], [51]. ACST led to the highest correlation between RCC1 and RCC2 datasets (60) as well as between BC1 and BC2 (66). The values of Spearman’s correlation obtained for SPIA and GSEA for colorectal cancer datasets are generally low (regardless of the number of pathways used) which indicates low repeatability of the results. Comparison of the results obtained with ACST and the mentioned methods showed that ACST may be a complementary method to the existing ones, with several favoring features.

### Conclusions

In this study we propose Analysis of Consistent Signal Transduction (ACST), a novel method for detection of deregulated signaling pathways using microarray gene expression data. ACST incorporates information about pathway topology, as well as about the position of every gene in each pathway. Using nine independent biological datasets, we have compared the performance of ACST with three methods commonly used to analyze signaling pathways in terms of biological significance (relevance of the results), as well as repeatability of the results among similar, yet independent, datasets. The obtained results led us to three conclusions. First, we demonstrate usefulness of predefined information of a pathway structure as well as genes functions in an analyzis of signaling pathways. Second, although not only ACST led to biologically meaningful results, we show that ACST is characterized by the highest repeatability among similar, yet independent, datasets. Third, we present that ACST is characterized by high specificity. In some cases it may result in loss of sensitivity, however, when false positives could not be easily excluded (e.g. verification of a novel function of a particular protein when nothing is known about the expected outcome) such characteristic is highly preferred.

## Supporting Information

File S1 An R code used to perform ACST.
(R) Click here for additional data file.

File S2 This document shows (i) list of analyzed pathways; (ii) results obtained with SPIA, BPA and GSEA; (iii) lists of eliminated.CEL files; (iv) correlations (with p{values) between results obtained for different datasets.
(XLS)

File S3 Fragments of pathways containing all consistent subgraphs of signifcantly deregulated pathways in both RCC datasets as well as in both BC datasets. Additionally, this file contains also table which contain a summary of results obtained with each of tested methods.
(PDF)
