# Supplementary material for: Identification of Pathway Deregulation – Gene Expression Based Analysis of Consistent Signal Transduction
Source: PLoS One. 2012 Jul 25;7(7):e41541. doi: 10.1371/journal.pone.0041541 (PMC3405133; doi:10.1371/journal.pone.0041541)
Supplement: File S3 — Fragments of pathways containing all consistent subgraphs of signifcantly deregulated pathways in both RCC datasets as well as in both BC datasets. Additionally, this file contains also table which contain a summary of results obtained with each of tested methods. (PDF) [file pone.0041541.s003.pdf]

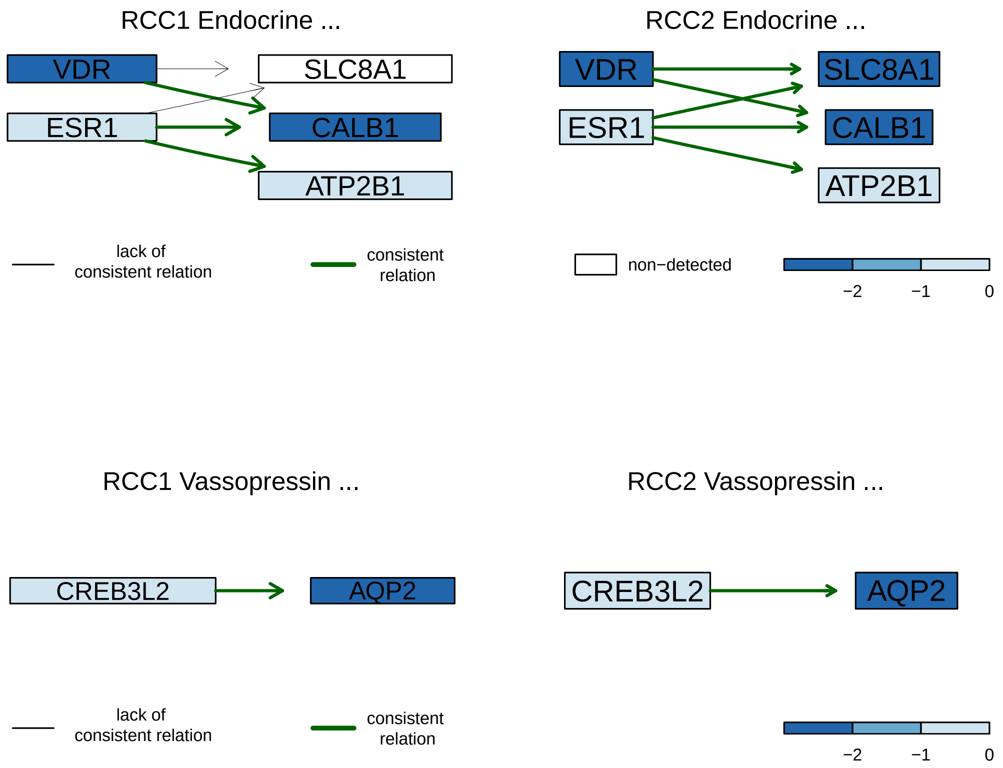

Figure 1: Fragments of pathways containing all consistent subgraphs of significantly deregulated pathways in both RCC datasets (All consistent subgraphs are presented, however, for a given pathway both presented fragments contain the same genes even if for a particular pathway these genes are not in consistent relations.). The blue color represents a higher expression in a control group. The color scale represents the magnitude of changes. The green arrows represent consistent relations. The white nodes represented genes that were not detected (due to type of microarray or mapping/filtration used)

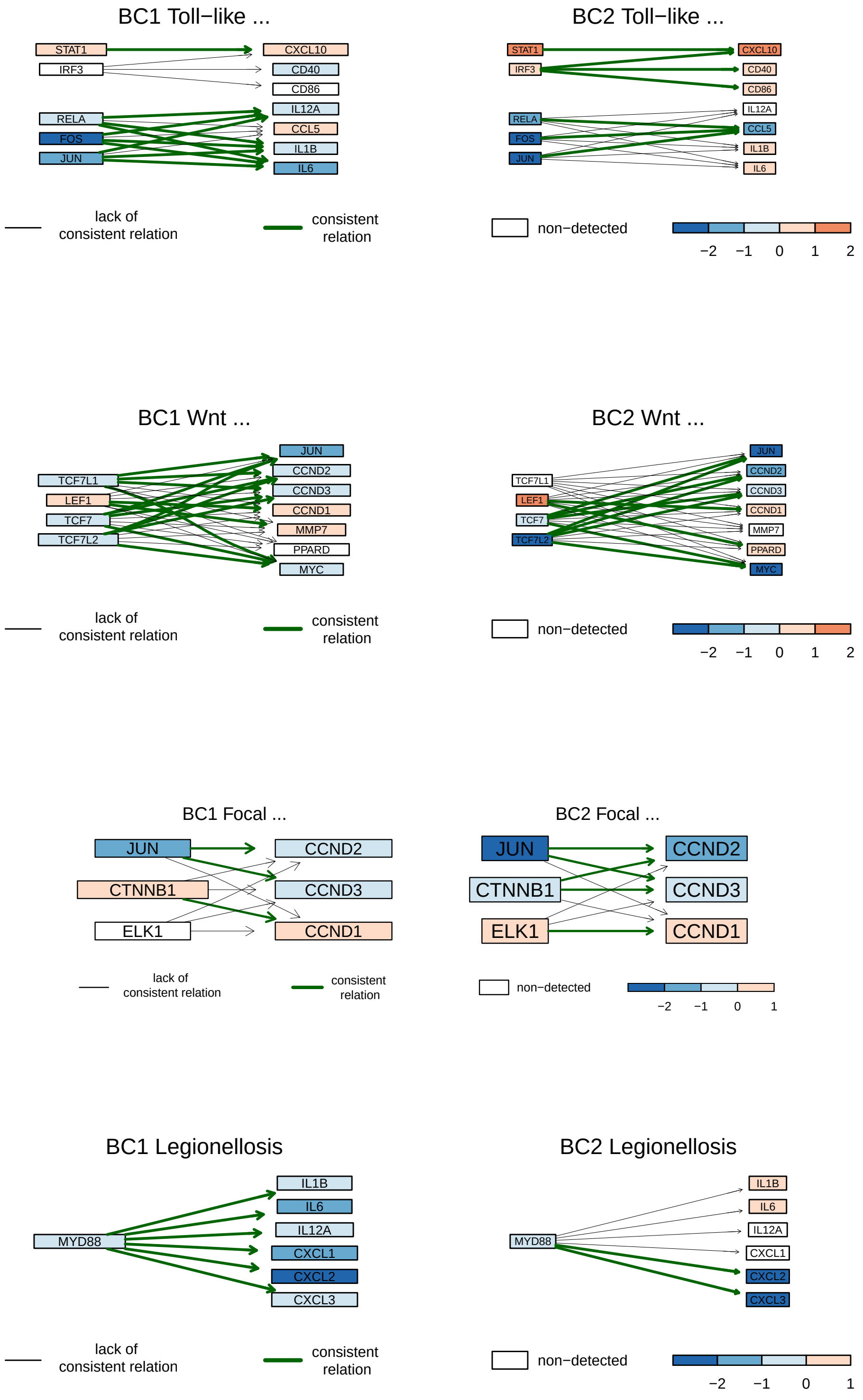

Figure 2: Fragments of pathways containing all consistent subgraphs of significantly deregulated pathways in both BC datasets (All consistent subgraphs are presented, however, for a given pathway both presented fragments contain the same genes even if for a particular pathway these genes are not in consistent relations.). The red color marks a higher expression in cancer samples, while the blue color represents a higher expression in a control group. The color scale represents the magnitude of changes. The green arrows represent consistent relations. The white nodes represented genes that were not detected (due to type of microarray or mapping/filtration used)

Table 1: **Repeatability of results**

a ACST

	CC1	CC2	CCms	CCad	RCC1	RCC2	BC1	BC2	VIN
CC1	8	3	2	2	0	0	5	4	0
CC2	41	9	3	7	0	0	2	3	1
CCms	19	56	6	4	0	0	1	3	0
CCad	19	59	37	13	1	1	1	3	1
RCC1	-1	8	32	30	3	2	0	0	0
RCC2	27	24	28	17	60	3	0	0	0
BC1	35	32	42	28	26	19	6	4	0
BC2	38	48	43	25	19	10	66	7	0
VIN	8	40	42	33	32	39	32	28	2

b BPA

	CC1	CC2	CCms	CCad	RCC1	RCC2	BC1	BC2	VIN
CC1	0 (29)	0 (12)	0 (11)	0 (13)	0 (11)	0 (6)	0 (20)	0 (5)	0 (24)
CC2	18 (50)	0 (14)	0 (8)	0 (8)	0 (8)	0 (5)	0 (11)	0 (4)	0 (13)
CCms	35 (42)	6 (47)	0 (18)	0 (8)	0 (10)	0 (6)	0 (12)	0 (4)	0 (14)
CCad	44 (50)	41 (46)	4 (46)	0 (19)	0 (9)	0 (5)	0 (14)	0 (6)	0 (14)
RCC1	-1 (47)	-15 (34)	15 (50)	32 (51)	0 (18)	0 (7)	0 (12)	0 (5)	0 (13)
RCC2	-19 (20)	22 (28)	24 (36)	9 (26)	0 (28)	10 (52)	0 (6)	0 (3)	0 (6)
BC1	14 (60)	-4 (41)	-26 (33)	25 (46)	0 (41)	8 (14)	0 (29)	0 (7)	0 (23)
BC2	13 (30)	32 (43)	26 (35)	14 (36)	17 (34)	16 (36)	5 (41)	0 (7)	0 (7)
VIN	11 (59)	8 (40)	-17 (30)	-14 (48)	12 (31)	6 (13)	19 (54)	18 (31)	0 (33)

c SPIA

	CC1	CC2	CCms	CCad	RCC1	RCC2	BC1	BC2	VIN
CC1	3 (2)	0 (0)	0 (0)	1 (1)	1 (2)	1 (2)	0 (0)	1 (2)	1 (2)
CC2	-7 (-13)	1 (2)	1 (2)	1 (2)	1 (1)	0 (0)	0 (0)	0 (0)	1 (2)
CCms	-9 (-2)	27 (38)	2 (3)	1 (2)	1 (2)	0 (1)	0 (0)	0 (1)	1 (2)
CCad	-2 (10)	42 (16)	41 (34)	6 (6)	2 (4)	1 (3)	0 (0)	2 (3)	2 (3)
RCC1	13 (18)	14 (-4)	35 (2)	5 (-27)	11 (16)	4 (12)	0 (0)	4 (9)	2 (3)
RCC2	4 (20)	16 (5)	33 (3)	9 (-15)	64 (75)	13 (13)	0 (0)	4 (8)	1 (2)
BC1	10 (8)	18 (6)	17 (-14)	-9 (-2)	-12 (22)	22 (15)	2 (1)	2 (1)	0 (0)
BC2	18 (19)	-3 (-3)	18 (-7)	17 (-1)	11 (39)	17 (40)	49 (53)	12 (12)	1 (2)
VIN	4 (-16)	14 (1)	41 (11)	47 (28)	29 (-29)	9 (-35)	-51 (-20)	38 (0)	3 (5)

d GSEA

	CC1	CC2	CCms	CCad	RCC1	RCC2	BC1	BC2	VIN
CC1	8 (67)	1 (27)	5 (34)	3 (33)	3 (36)	2 (35)	6 (17)	4 (5)	2 (21)
CC2	9 (-4)	1 (41)	3 (21)	2 (21)	3 (23)	3 (22)	0 (10)	1 (1)	4 (15)
CCms	23 (-5)	32 (-7)	8 (54)	6 (31)	7 (29)	6 (28)	6 (15)	3 (2)	3 (23)
CCad	42 (8)	54 (12)	49 (20)	6 (53)	6 (27)	5 (26)	4 (11)	4 (4)	3 (26)
RCC1	12 (3)	11 (6)	37 (-5)	31 (-8)	8 (54)	8 (26)	4 (10)	4 (6)	4 (21)
RCC2	20 (6)	29 (3)	39 (5)	41 (3)	73 (-4)	6 (51)	4 (13)	3 (4)	4 (17)
BC1	17 (3)	-5 (-5)	26 (21)	-10 (3)	-7 (-19)	1 (-1)	12 (26)	4 (2)	1 (11)
BC2	10 (10)	-3 (10)	-6 (5)	3 (8)	3 (4)	10 (5)	27 (-7)	11 (6)	2 (5)
VIN	30 (-17)	80 (2)	27 (-6)	61 (16)	12 (-7)	31 (-10)	8 (5)	14 (10)	3 (47)

The tables contain a summary of results obtained with each of tested methods. Each table represents results for a different method and is constructed as follows: let i and j stand respectively for row and column number of a given table A. Then if $i = j$ element a_{ij} shows a number significantly altered pathways indicated by a given method. If $i < j$ (above diagonal) the element a_{ij} shows a number of significantly altered pathways indicated in $i - th$ and $j - th$ datasets. If $i - th$ and $j - th$ datasets if $i > j$ (below diagonal) the element a_{ij} shows a Spearman correlations (multiplied by 100) of nominal p–values computed for $i - th$ and $j - th$ datasets. The diagonals of each table were highlighted in gold. Using light and dark green we highlighted the results for similar datasets. Table A presents results for ACST, B for BPA, C for SPIA and D for GSEA. Tables for BPA, SPIA and GSEA show values computed for signaling pathway used by default (in brackets) and only for pathways used by ACST.
